# Exploring metacognitions in health anxiety and chronic pain: a cross-sectional survey

**DOI:** 10.1186/s40359-020-00455-9

**Published:** 2020-08-07

**Authors:** Geoffrey S. Rachor, Alexander M. Penney

**Affiliations:** Department of Psychology, MacEwan University, 10700-104 Avenue, 6-329, Edmonton, AB T5J-4S2 Canada

**Keywords:** Chronic pain, Disability, Health anxiety, Metacognitions, Metacognitions about health

## Abstract

**Background:**

The occurrence of health anxiety (HA) in chronic pain is associated with adverse outcomes. As such, it is important to identify constructs that might influence HA and pain-related outcomes. Metacognitions are an emerging area of interest in both HA and chronic pain, but the relationship between the three factors has not been extensively examined. The current study sought to examine the role of metacognitions about health in HA and pain-related outcomes in chronic pain.

**Methods:**

This study utilized a cross-sectional design. Undergraduate students with self-reported chronic pain (*n* = 179) completed online measures of HA, pain intensity, pain disability, and metacognitions about health.

**Results:**

Regression analyses indicated that both metacognitions about biased thinking and that thoughts are uncontrollable predicted HA in chronic pain, while only metacognitions about biased thinking predicted pain-related disability beyond pain intensity.

**Conclusion:**

Results demonstrate that HA and pain-related disability are not associated when taking metacognitions about health into account, suggesting that metacognitions about health at least partially account for the relationship between the two. Further, results suggest that metacognitions about biased thinking may independently influence HA and pain-related disability within chronic pain.

## Background

Chronic pain is a prevalent condition among university-aged adults [1]. Research has demonstrated that the prevalence of chronic pain in young adults ranges between 10% [2] to 17% [1]. Health anxiety (HA) often complicates outcomes for individuals with chronic pain. The occurrence of HA in chronic pain is estimated to be as high as 51% [3], and is associated with pain-related disability and maladaptive pain behaviours [4, 5]. As there is conceptual overlap between cognitive constructs in both HA and chronic pain, examining HA constructs within pain samples may enhance our understanding of the relationship between the two [5, 6]. For example, outcomes in HA are influenced by selective attention to illness information, which prompt dysfunctional assumptions about health and influence catastrophic beliefs that maintain HA [3, 7]. Similarly, pain-related disability is influenced by catastrophic misinterpretations of somatic sensations [8] and attentional bias to pain-related information [9].

Recently, researchers have been examining broader cognitive constructs, namely metacognitions, in both HA and chronic pain outcomes. Metacognitions are higher-order beliefs that individuals have about their own thinking and how it should be controlled [10, 11]. These beliefs are widely regarded as important factors contributing to the escalation and maintenance of worry [10, 11]. According to the Self-Regulatory Executive Function model of emotional disorders [12], excessive worry is activated and maintained by metacognitions [13, 14].

Research in HA indicates that metacognitions predict HA alongside, and often beyond, more specific cognitive factors, such as attentional bias towards health-related information and catastrophizing [7, 15–19]. In pain research, there has been a focus on how metacognitions are associated with pain catastrophizing, and how both constructs influence pain outcomes [11, 20–22]. For example, researchers have found metacognitions to account for the relationship between pain catastrophizing and pain-related behaviours, such as avoidance and help-seeking [11, 20]. Evidently, the Self-Regulatory Executive Function model presents unique implications for research in HA and chronic pain, as maladaptive beliefs in HA may reflect broader worries about thoughts, rather than more specific worries about illness [15].

Despite researchers having identified important psychological factors that contribute to pain outcomes, current evidence of psychological interventions targeting these specific factors, such as pain catastrophizing, have yielded inconsistent results [23]. As such, it is important for researchers to further attempt to delineate the relationships between psychological factors that predict pain outcomes. Such efforts may enhance our theoretical understanding of the psychological processes involved in predicting pain outcomes and may highlight novel areas for further research. As no research to date has examined metacognitions and HA together in chronic pain, this study sought to examine whether metacognitions about health predict HA and pain-related outcomes in a sample of university students with self-reported chronic pain. The self-report of pain is considered the gold-standard in pain assessment [24], suggesting there is clinical relevance to studying pain-related constructs in non-clinical samples, such as in the current study.

## Methods

### Participants

A convenience sample of undergraduate students with self-reported chronic pain (*N* = 191) self-selected to participate in this study. This sample was primarily recruited from a large research pool of undergraduate psychology students. Undergraduate psychology students created an account through the university’s online research management system, after which they were prompted to complete a series of questionnaires to determine their eligibility for various studies. As part of this screening process, potential participants completed two screening items to be eligible for the present study: ‘Do you experience chronic pain?’, and ‘Do you experience significant pain on a regular (e.g., daily or every other day) basis?’. Individuals must have answered yes to at least one of the screening items to be eligible for our study. Upon starting the study, participants also indicated the length, in months, of their pain condition. Any participant indicating a condition lasting less than 3 months, or that did not provide an answer, was excluded from analyses (*n* = 12). The final sample (*n* = 179) was primarily Caucasian (75.50%) and was comprised of females (81.56%), with a mean age of 22.18 years (*SD* = 5.41).

### Measures

#### Chronic pain grade scale (CPGS) [25]

The CPGS [25] is a seven-item self-report questionnaire consisting of three subscales: a) pain intensity, b) disability days, and c) pain disability. The current study used the CPGS pain intensity and pain disability subscales. Each of these subscales consist of three 11-point Likert-type scale items, with total scores ranging from 0 to 100, where individuals rate the intensity of their pain ‘right now’, ‘on average’, and ‘at its worst’, as well as the extent to which pain interferes with their daily activities, social and recreational activities, and work or school activities. Total scores are derived by averaging the sum of individual items comprising each subscale and multiplying this score by 10. The CPGS has demonstrated strong psychometric properties and has been validated for use in non-clinical samples [26].

#### Short health anxiety inventory (SHAI) [27]

The SHAI [27] is an 18-item self-report measure of HA. Total scores on the SHAI range from 0 to 54, with higher scores indicating elevated HA. The SHAI possesses strong psychometric properties and is valid for use in non-clinical samples [27, 28].

#### Metacognitions about health questionnaire (MCQ-HA) [29]

The MCQ-HA [29] is a 14-item self-report measure of metacognitions about health. The MCQ-HA contains three subscales: beliefs about biased thinking (MCQ-HAB), beliefs that thoughts can cause illness (MCQ-HAC), and beliefs that thoughts are uncontrollable (MCQ-HAU). MCQ-HAB subscale scores can range from 5 to 25 and reflect beliefs such as “thinking the worst about my symptoms will keep me safe”. MCQ-HAC subscale scores can range from 5 to 25 and reflect beliefs such as “thinking negatively can increase my chances for disease”. MCQ-HAU subscale scores can range from 4 to 20 and reflect beliefs such as “dwelling on thoughts of illness is uncontrollable”. The MCQ-HA has demonstrated strong psychometric properties [29].

### Procedure

This study received approval from the university’s research ethics board. Potential participants were recruited either through the psychology department’s research participation pool, or by responding to recruitment posters on campus. All participants completed an online informed consent procedure as well as a demographics questionnaire, after which they completed the CPGS, SHAI, and MCQ-HA, sequentially. Upon completion, participants received either research participation credits, or were entered into a draw for a $25 gift card.

### Statistical analyses

Prior to data analysis, skewness and kurtosis were examined for scores on the SHAI, CPGS pain intensity and pain disability subscales, and MCQ-HAB, MCQ-HAC, and MCQ-HAU subscales to determine whether the data met assumptions for statistical testing. Pearson product-moment correlations were then calculated between scores on these scales, with alpha set to .05. To identify which variables predict HA and pain-related outcomes, hierarchical regression analyses were conducted with alpha set to .05, with established predictors entered in the first step of the models, and metacognitions about health entered in the second step of the models. To further identify the strongest predictors of HA and pain-related outcomes, forward stepwise regression analyses were then conducted. Criteria for forward stepwise regression modelling in the current study were set as probability of *F* to be entered into the model ≤ .05, and probability of *F* to be removed from the model as ≥ .10. For all regression analyses, only predictor variables that were significant in the correlation analyses were entered.

## Results

Means, standard deviations, Cronbach’s alpha, and Pearson product-moment correlations for all scale and subscale scores are reported in Table 1. An examination of skewness and kurtosis indicated MCQ-HAB to violate assumptions of normality for statistical testing, 1.76 (*SE* = 0.182) and 2.41 (*SE* = 0.362), respectively. An inverse transformation of MCQ-HAB was computed, bringing values of skewness (−.76, *SE* = 0.182) and kurtosis (−.51, *SE* = 0.362) within acceptable limits [30]. This inverse transformation of MCQ-HAB was used for all analyses in the current study. Due to the inverse transformation, all relationships with the MCQ-HAB scores are interpreted in the opposite direction (e.g., a negative correlation between MCQ-HAB scores and SHAI scores indicates that as the MCQ-HAB scores increase, so do SHAI scores). All other variables were within acceptable ranges for skewness and kurtosis [30]. Correlation analyses indicated that SHAI scores correlated with CPGS pain disability scores, but not with pain intensity scores. SHAI scores also correlated with MCQ-HAB, MCQ-HAC, and MCQ-HAU scores. CPGS pain intensity and pain disability scores correlated with one another, and both correlated with MCQ-HAB and MCQ-HAU subscale scores. MCQ-HAC scores did not correlate with either pain intensity or pain disability scores.

**Table 1:** Means, standard deviations, Cronbach’s alpha, and Pearson product-moment correlations

	MEAN	SD	ALPHA	1	2	3	4	5
1. SHAI	20.27	7.46	.86	–				
2. CPGS-Intensity	57.85	15.37	.67	.12	–			
3. CPGS-Disability	44.47	23.22	.87	.21^**^	.44^***^	–		
4. MCQ-HAB	6.87	2.54	.79	−.49 ^***^	.16^*^	−.29 ^***^	–	
5. MCQ-HAC	9.99	3.87	.86	.28 ^***^	.02	−.01	−.27 ^***^	–
6. MCQ-HAU	7.51	2.54	.63	.57 ^***^	.19^*^	.25 ^**^	−.49 ^***^	.29 ^***^

*SHAI* Short Health Anxiety Inventory, *CPGS-Intensity* Pain Intensity, *CPGS-Disability* Pain Disability, *MCQ-HAB* Beliefs about Biased Thinking, *MCQ-HAC* Beliefs that Thoughts can Cause Illness, *MCQ-HAU* Beliefs that Thoughts are Uncontrollable

^*^p < .05. ^**^p < .01. ^***^p < .001

Hierarchical regression analyses were conducted with either SHAI, CPGS pain intensity, or CPGS pain disability subscale scores entered as the dependent variable, with the other two variables entered on step one of each model. For example, with SHAI scores entered as the dependent variable, CPGS pain intensity and CPGS pain disability scores were entered in step one. In order to examine the unique contributions of metacognitions about health on SHAI, CPGS pain intensity, and CPGS pain disability subscale scores, MCQ-HAB, MCQ-HAC, and MCQ-HAU subscale scores were entered in step two of the models. For all analyses, only those variables that were significant in the correlation analyses were entered into regression models. Results of these hierarchical regression analyses are reported in Table 2.

**Table 2:** Results of hierarchical regression analyses

Variable	*R*	*R*^*2*^	*R*^*2*^ Change	*t*	*pr*
DV: SHAI					
Step 1	.213	.045	.045**		
CPGS-DISABILITY				2.89**	.21**
Step 2	.622	.386	.341***		
CPGS-DISABILITY				0.63	.05
MCQ-HAB				−3.50**	−.26**
MCQ-HAC				1.46	.11
MCQ-HAU				5.79***	.40***
DV: CPGS-INTENSITY					
Step 1	.435	.189	.189***		
CPGS-DISABILITY				6.38***	.44***
Step 2	.443	.196	.007		
CPGS-DISABILITY				5.71***	.40***
MCQ-HAB				0.00	.00
MCQ-HAU				1.09	.08
DV: CPGS-DISABILITY					
Step 1	.468	.219	.219***		
SHAI				2.55*	.19*
CPGS-INTENSITY				6.08***	.42***
Step 2	.498	.248	.029*		
SHAI				0.53	.04
CPGS-INTENSITY				5.69***	.40***
MCQ-HAB				−2.03*	−.15*
MCQ-HAU				0.98	.08

*pr* Partial correlation, *SHAI* Short Health Anxiety Inventory, *CPGS-Intensity* Pain Intensity, *CPGS-Disability* Pain Disability, MCQ-HAB Beliefs about Biased Thinking, *MCQ-HAC* Beliefs that Thoughts can Cause Illness, *MCQ-HAU* Beliefs that Thoughts are Uncontrollable

^*^p < .05. ^**^p < .01. ^***^p < .001

With SHAI scores entered as the dependent variable, results of the hierarchical regression analysis indicated that MCQ-HAB and MCQ-HAU subscale scores significantly predicted SHAI scores while controlling for CPGS pain disability scores in step one of the model. MCQ-HAC scores did not significantly predict SHAI scores beyond CPGS pain disability scores. With CPGS pain intensity scores entered as the dependent variable, it was found that only CPGS pain disability scores predicted CPGS pain intensity scores. MCQ-HAB and MCQ-HAU subscale scores did not significantly predict CPGS pain intensity scores while controlling for CPGS pain disability scores. Lastly, with CPGS pain disability scores entered as the dependent variable, MCQ-HAB subscale scores significantly predicted CPGS pain disability scores while controlling for SHAI scores and CPGS pain intensity subscale scores in step one of the model. MCQ-HAU subscale scores did not significantly predict CPGS pain disability scores beyond SHAI and CPGS pain intensity scores.

To identify the strongest predictors of SHAI, CPGS pain intensity, and CPGS pain disability subscale scores, the hierarchical regression analyses were supplemented with forward stepwise regression analyses. Forward stepwise regression analyses were conducted with SHAI, CPGS pain intensity, or CPGS pain disability subscale scores entered as the dependent variable, while all remaining potential predictor variables were entered as independent variables. For all analyses, only those variables that were significant in the correlation analyses were entered into regression models. In all outlined models that follow, only significant results are reported.

With SHAI scores entered as the dependent variable, only MCQ-HAB and MCQ-HAU emerged as significant predictors. MCQ-HAU scores emerged as the strongest predictor of SHAI scores, *β* = .43, *t*(173) = 6.31, *p* < .001, while MCQ-HAB scores added additional predictive value, *β* = −.27, *t*(173) = − 3.98, *p* < .001. The full regression model resulted in a two-step model whereby MCQ-HAU and MCQ-HAB scores together accounted for 37.1% of the variance in SHAI scores, *F*(2, 173) = 53.19, *p* < .001. Individually, MCQ-HAU subscale scores accounted for 32.2% of the full model, *F*(2, 173) = 83.48, *p* < .001, while MCQ-HAB scores accounted for an additional 5.6%, *∆ F*(2, 173) = 15.85, *p* < .001.

With CPGS pain intensity scores as the dependent variable, the forward stepwise regression analysis indicated that only scores on the CPGS pain disability subscale were predictive, *β* = .44, *t*(173) = 6.38, *p* < .001. CPGS pain disability subscale scores accounted for 18.9% of the variance in pain intensity scores, *F*(1, 174) = 40.67, *p* < .001.

Lastly, with CPGS pain disability scores as the dependent variable, the forward stepwise regression analysis indicated that only scores on MCQ-HAB significantly predicted pain disability scores beyond CPGS pain intensity. CPGS pain intensity scores emerged as the strongest predictor of pain disability, *β* = .40, *t*(173) = 5.93, *p* < .001, while MCQ-HAB scores added additional predictive value beyond that of pain intensity, *β* = −.23, *t*(173) = − 3.34, *p* = .001. The full regression model resulted in a two-step model whereby CPGS pain intensity and MCQ-HAB scores together accounted for 23.0% of the variance in pain disability scores, *F*(2, 173) = 27.16, *p* < .001. Individually, CPGS pain intensity subscale scores accounted for 18.9% of the full model, *F*(2, 173) = 40.67, *p* < .001, while MCQ-HAB scores accounted for 5.0%, *∆ F*(2, 173) = 11.26, *p* = .001.

## Discussion

To our knowledge, this is the first study to examine metacognitions about health in the relationship between HA and chronic pain. Results of the current study lend support to the idea that examining pain variables within the context of HA may enhance our understanding of the relationship between the two [5]. Specifically, we found that metacognitions about biased thinking and metacognitions that thoughts are uncontrollable predicted HA in an undergraduate student sample with self-reported chronic pain, even when controlling for pain-related disability. We also found that both pain intensity and metacognitions about biased thinking independently predicted pain-related disability. However, only pain disability was associated with pain intensity. Notably, results demonstrated a non-significant relationship between HA and pain-related disability when taking metacognitions about health into account. With only metacognitions about biased thinking predicting both HA and pain-related disability, these results highlight metacognitions about biased thinking as an important possible link between the two constructs.

The present findings suggest that metacognitions about health, particularly metacognitions about biased thinking, are an important consideration in co-occurring HA and chronic pain. This claim is strengthened by the finding that HA and pain-related disability were not predictive of one another beyond these metacognitions. Taken together, these findings would suggest that within the Self-Regulatory Executive Function model of emotional disorders [12], that metacognitions about biased thinking and that thoughts are uncontrollable may drive the relationship between co-occurring HA and chronic pain. Here, anxiety would result from the activation of metacognitions about biased thinking and about the uncontrollability of thoughts, which would perpetuate maladaptive thought- and behavioural-control strategies [13, 14], such as attentional bias towards health information and somatic sensations [7, 11] and catastrophic thinking [11, 16].

Greater emphasis on the role of metacognitions about biased thinking in co-occurring HA and chronic pain may be warranted, as these metacognitions appeared to link HA and pain-related disability. This suggests that pain-related disability may be partially due to the dysfunctional metacognitions individuals hold regarding how they should think about their overall health, in addition to their beliefs specifically regarding their pain condition. The results of the current study might suggest that metacognitions about biased thinking could result in an attentional bias towards symptoms, thereby resulting in increased catastrophizing and maladaptive behaviours that contribute to both elevated HA and pain-related disability, such as avoidance and safety-seeking behaviours [3, 7, 11]. Clinical implications of our study suggest that Metacognitive Therapy [14] is a potentially viable treatment option for individuals with chronic pain, and that specifically targeting metacognitions about biased thinking might help improve pain-related disability outcomes.

### Limitations

This study has important limitations that warrant acknowledgement. First, our use of a non-clinical convenience sample may limit the generalizability of our results beyond student populations [31], as we were unable to verify pain diagnoses and did not collect information on the various types of pain conditions experienced by participants. However, the current study screened participants for self-reported chronic pain, thereby suggesting some degree of external validity. Further, while reliance on self-report measures may limit the results of the current study, all measures that were utilized have demonstrated strong psychometric properties and have been validated for use in non-clinical samples [26–29].

As the current study did not include measures of other related factors, such as somatosensory amplification and catastrophizing [8, 16, 20–22], researchers may wish to further examine the role of metacognitions about health while also taking these constructs into account. Additionally, as the data obtained in the current study was cross-sectional in nature and collected at only one point in time, we were unable to examine the causal connection between variables. Future research may seek to obtain longitudinal data in order to delineate the relationship between metacognitions about health and HA and pain-related outcomes.

Lastly, the use of forward stepwise regression techniques has been criticized for over-simplifying results [32]. It should be noted that the purpose of using forward stepwise regression techniques in our study was to supplement hierarchical regression analyses, with the purpose of identifying the strongest predictors of HA and pain-related outcomes.

## Conclusions

Results of the current study support previous findings that metacognitions are an important consideration in both HA [15–19] and chronic pain [10, 20–22], and extend this to the application of metacognitions about health in co-occurring HA and chronic pain. Specifically, results indicate that metacognitions about health may independently influence HA and pain-related outcomes, and suggest that both HA and pain-related disability may share an underlying etiological factor, namely metacognitions about biased thinking. Further evidence is needed in order to clearly delineate the relationships between metacognitions about health, HA, and pain-related outcomes. Metacognitions about health warrant further investigation in a clinical sample with chronic pain, regardless of the occurrence of HA. Further examination of metacognitions about health in HA is also warranted.

## Data Availability

The datasets generated and/or analysed during the current study are not publicly available due to ethics restrictions but are available from the corresponding author, AP, on reasonable request.

## Abbreviations

CPGS: Chronic Pain Grade Scale
HA: Health Anxiety
MCQ-HA: Metacognitions about Health Questionnaire
MCQ-HAB: Belief about Biased Thinking
MCQ-HAC: Beliefs that Thoughts can Cause Illness
MCQ-HAU: Beliefs that Thoughts are Uncontrollable
SHAI: Short Health Anxiety Inventory
